# Transformation Paths from Cubic to Low-Symmetry Structures in Heusler Ni_2_MnGa Compound

**DOI:** 10.1038/s41598-018-25598-z

**Published:** 2018-05-08

**Authors:** Martin Zelený, Ladislav Straka, Alexei Sozinov, Oleg Heczko

**Affiliations:** 1 NETME Centre, Faculty of Mechanical Engineering, Institute of Materials Science and Engineering, Brno University of Technology, Brno, CZ-61669 Czech Republic; 2 Faculty of Mathematics and Physics, Charles University, Prague, CZ-12116 Czech Republic; 3 Institute of Physics, Czech Academy of Sciences, Prague, CZ-18221 Czech Republic; 4 Material Physics Laboratory, Lappeenranta University of Technology, Savonlinna, FI-57170 Finland

## Abstract

In order to explain the formation of low-temperature phases in stoichiometric Ni_2_MnGa magnetic shape memory alloy, we investigate the phase transformation paths from cubic austenite with Heusler structure to low-symmetry martensitic structures. We used *ab initio* calculations combined with the generalized solid state nudged elastic band method to determine the minimum energy path and corresponding changes in crystal lattice. The four-, five-, and seven-layered modulated phases of martensite (4O, 10M, and 14M) are built as the relaxed nanotwinned non-modulated (NM) phase. Despite having a total energy larger than the other martensitic phases, the 10M phase will spontaneously form at 0 K, because there is no energy barrier on the path and the energy decreases with a large negative slope. Moreover, a similar negative slope in the beginning of path is found also for the transformation to the 6M premartensite, which appears as a local minimum on the path leading further to 10M martensite. Transformation paths to other structures exhibit more or less significant barriers in the beginning hindering such a transformation from austenite. These findings correspond to experiment and demonstrates that the kinetics of the transformation is decisive for the selection of the particular low-symmetry structure.

## Introduction

The multiferroic Heusler Ni-Mn-Ga system is the prototype magnetic shape memory (MSM) material^1^. It can exhibit the so-called magnetic shape memory effect or magnetically-induced reorientation (MIR) with up to 12% strain in all of its three most common martensitic phases: in five-layered modulated tetragonal martensite (10M), in seven-layered modulated orthorhombic martensite (14M), and in non-modulated (NM) tetragonal martensite with a slight modification of the Ni-Mn-Ga composition by adding few at. % of Co and Cu.^2,3^. The existence of a large MSM effect in two or three different martensites of the same system has not been reported in any other material, which justifies the strong research focus on this particular system. Simultaneously, it provides a unique insight into the universal principles and mechanisms related to the magnetic shape memory phenomenon by comparison between the individual martensites. This is very important for the generalization of the MSM phenomenon and transferring the knowledge to other MSM alloys with similar or different types of martensites^4,5^.

The prerequisite for the MSM effect and the related extraordinary magnetomechanical properties of MSM materials is the low-symmetry martensitic phase with a large magnetic anisotropy and twinned microstructure with low twinning stress^1^. During a martensitic transformation, the low-symmetry martensite appears due to its lower free energy in comparison with the high temperature parent cubic phase, called austenite. It has been suggested that the large variety of low-temperature martensitic phases originates in the complex electronic structure resulting in competition between the kinetics driven by the softening of the TA_2_ [ξξ0] phonon branch in austenite^6–9^ with the band Jahn–Teller effect, stabilizing the martensite^10–12^.

*Ab initio* calculations predict that the experimentally known martensitic phases (NM, 14M, 10M) are metastable at 0 K. The ground state of Ni_2_MnGa has been predicted to be a 4O martensitic phase at 0 K^13^, which, however, was not found in experiment. The inconsistency between the theory and experiment motivates us to study also the kinetic aspects of the transformations in addition to the previous thermodynamic approach^13–16^.

In this paper, we compare the phase transformation paths and corresponding energy barriers between the austenite and the individual types of martensites in stoichiometric Ni_2_MnGa. To determine these pathways, we extend the *ab initio* calculations based on spin-polarized Density Functional Theory by the Generalized Solid State Nudged Elastic Band (G-SSNEB) method^17^. This provides a way to find minimum energy paths just with the knowledge of the starting and final lattice only, and to identify the effects responsible for the formation of a particular martensitic structure. The comparison of the paths explains why the transformation to the 4O structure is replaced by the transformation to 10M. This transformation is driven by the phonon soft mode of austenite in the early stage and by the Jahn–Teller effect in a later stage.

## Results

**Figure 1:**
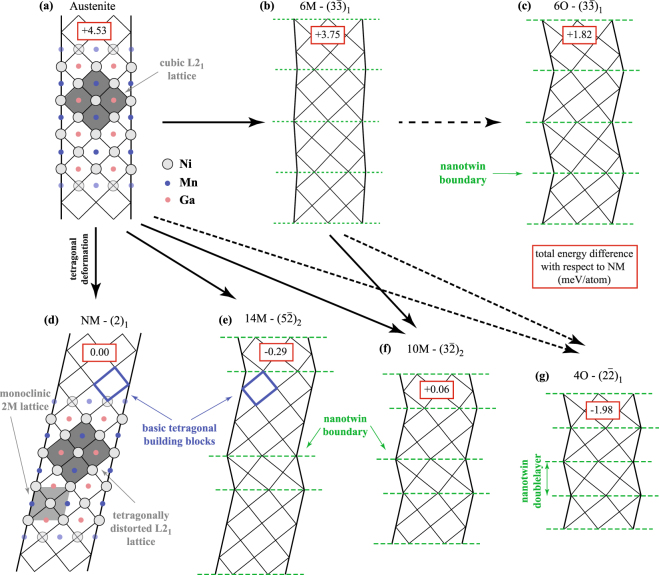
Schematic illustration of the austenite A (**a**), premartensite 6M (**b**) and martensite structures: 6O (**c**), NM (**d**), 14M (**e**), 10M (**f**) and 4O (**g**) in Ni-Mn-Ga system (6O is present for the purpose of comparison, it has not been reported for Ni-Mn-Ga). Gray filling in A and NM identify the original and tetragonally distorted L2_1_ cell and monoclinic 2M cell, respectively; basic tetragonal building blocks are marked with blue in (**d**) and (**e**); the green dashed lines mark nanotwin boundaries; numbers in red boxes correspond to total energy differences in meV/atom relatively to NM martensite.

Using the idea of nanotwinning^18,19^ arising from the adaptive martensite theory^20^, all known martensitic structures can be constructed from two types of NM building blocks with alternating Mn or Ga in the center. The building block is one-eighth of the volume of the tetragonal cell derived from the L2_1_ cubic cell of austenite^21^. Such constructed NM, 14M, 10M structures and theoretically predicted 4O structure are illustrated in Fig. 1 together with cubic austenite, 6M premartensite and the 6M-related hypothetical 6O structure (building blocks marked with blue in Fig. 1(d) and (e) and L2_1_ cubic cell marked with gray in Fig. 1(a)).

The experimentally observed 10M and 14M modulated martensites contain double layers, which have a stabilizing effect on the given structure due to the negative contribution of the nanotwin boundary–boundary interactions to the total energy^13^. The double layer can be regarded as built of the primitive monoclinic cells of the NM structure, often called 2M^22^ (marked with light gray in Fig. 1(d)). The double layers alternate with triple layers or pentuple layers in 10M and 14M, respectively. The multiple layers can be seen as nanotwins of the NM martensite with (101) plane as a twin boundary plane^18,19^, forming the modulated structure by their periodic arrangement, denoted by ${(3\bar{2})}_{2}$ and ${(5\bar{2})}_{2}$. The theoretically predicted 4O martensite consists only of alternating double layers and is denoted by ${(2\bar{2})}_{1}$.

The modulated 6M premartensite with cubic symmetry appears just above martensitic transformation temperature in near stoichiometric alloys as a precursor of the martensitic transformation^23^. The 6O structure was constructed artificially from triple layers for the purpose of discussion and comparison. The 6O $(\ldots 3\bar{3}3\bar{3}\ldots )$ and 4O $(\ldots 2\bar{2}2\bar{2}\ldots )$ structures have never been observed experimentally, but the local sequences of two oppositely oriented triple or double layers, i.e., $(\ldots 2\bar{3}2\bar{3}3\bar{2}3\bar{2}\ldots )$, $(\ldots 3\bar{2}3\bar{2}2\bar{3}2\bar{3}\ldots )$ were suggested to occur in 10M martensite. They originate from the inversion of the ${(3\bar{2})}_{2}$ stacking sequence, which form the {110} compound or *a*/*b* twin boundaries at mesoscopic scale^24,25^.

**Figure 2:**
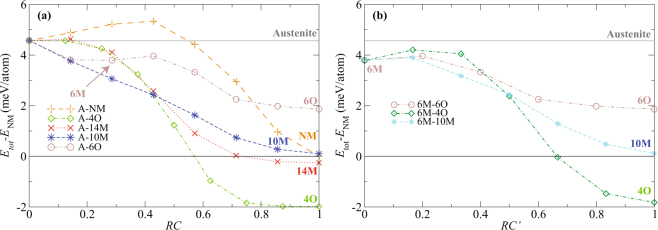
Calculated minimum energy path (MEP) of Ni_2_MnGa along the reaction coordinates, *RC′* and *RC*, for the transformation starting from austenite (**a**) and from 6M premartensite (**b**) (*RC* = 0: austenite; *RC*′ = 0: 6M premartensite; *RC* = 1, *RC*′ = 1: fully transformed martensite). All energies are relative to the total energy of NM martensite.

The calculated minimum energy paths (MEP) at 0 K of all martensites are shown in Fig. 2(a), with the total energy given relative to the energy of NM structure. Since we deal with several different lattices, we need a universal coordinate, which is independent of the particular lattice geometry or arrangement of atoms along the transformation path. The reaction coordinate, *RC*, used in Fig. 2, is an abstract coordinate which universally defines the progress of transformation between austenite (*RC* = 0) and fully transformed martensite (*RC* = 1). All the energies in the figure are fully relaxed with respect to the transformation path involving both atomic and unit-cell degrees of freedom. For the transformation between austenite (A) and NM martensite, the obtained barrier and transformation path are identical to those already described in the literature^6,7,9,26^ without using the G-SSNEB, which confirms the validity of our approach. The A–NM path corresponds to tetragonal deformation realized by pure shear^27^. For the A–4O and A–14M transformations, there is an energy barrier in the beginning of the transformation at RC ≈ 0.15.

For the A–6O transformation the energy steeply decreases in the beginning and the lattice becomes tetragonal. Then local minimum appears at RC ≈ 0.29, which corresponds to 6M premartensite and the cubic symmetry of the lattice is restored (Fig. 2(a)). Overcoming the local minimum, the transformation continues to a tetragonal 6O structure. This hypothetical structure exhibits the highest total energy of all the martensites included in this study.

Surprisingly, the A–10M transformation exhibits no barrier and the energy monotonically decreases from A to 10M with the largest initial gradient. Thus, this is the most favorable path, and the austenite will spontaneously transform to 10M martensite at 0 K. In fact, even the simple linear interpolation of structures between L2_1_ austenite and 10M martensite directly provides a barrierless energy path. The relaxation of these linearly interpolated structures by the G-SSNEB algorithm just further increases the energy gradient. This is different from other martensites, where the path obtained by linear interpolation always contains a significant barrier which can be decreased–but not eliminated–by finding an MEP with the help of G-SSNEB.

Since the experimentally observed transformation is A → 6M → 10M for stoichiometric Ni_2_MnGa, we also calculate the 6M–10M and 6M–4O transformation paths. They are displayed in Fig. 2(b) together with the 6M–6O transformation where *RC′* = 0 corresponds to *RC* ≈ 0.29 in Fig. 2(a). All paths exhibit barriers in the beginning of transformation. Again, the lowest barrier can be found along the 6M–10M transformation, which makes this path energetically the most favorable. On the other hand, the 6M–4O transformation to the energetically most preferred structure 4O exhibits the highest barrier, which prevents this transformation.

**Figure 3:**
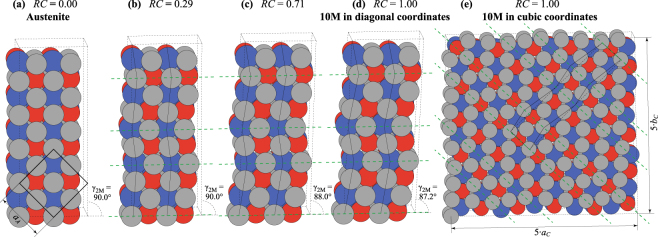
(**a**–**d**) Selected images from the calculated transformation path A–10M in diagonal (2M) coordinates. The reaction coordinate is indicated above the images (*RC* = 0: austenite; *RC* = 1: fully transformed 10M martensite). The L2_1_ lattice is marked with thick black lines in (**a**). (**e**) The cell of 10M martensite in cubic coordinates. Dashed black lines correspond to the same structure in diagonal (2M) coordinates. Green dashed lines correspond to nanotwin boundaries.

Before we start explaining the structural changes in the lattices along the transformation paths, let us briefly introduce two descriptions of the modulated structures commonly used in literature^21^. The 10M, 14M and NM structures in Fig. 1 are based on the nanotwinning concept. The primitive monoclinic cell is constructed by assuming a stacking sequence of the 2M cells. The same description is used in Fig. 3(a)–(d), which shows four example snapshots of structure from the A–10M path. This description in diagonal coordinates is widely used for computer simulations, because it allows keeping the smallest possible atomic bases. A common alternative description of the 10M and 14M structures is based on cubic coordinates derived from the L2_1_ lattice. This needs a much larger unit cell to encompass the full modulation period, as illustrated for the 10M structure in Fig. 3(e). The lattice parameters *a*_*C*_ and *b*_*C*_ are normalized by the number of layers in the modulation period to keep them comparable with the cubic structure of the austenite.

**Figure 4:**
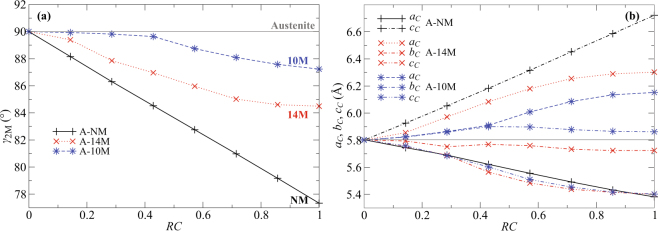
Monoclinic angle *γ*_2*M*_ (**a**) and lattice parameters *a*_*C*_, *b*_*C*_, *c*_*C*_ in cubic coordinates (**b**) as functions of reaction coordinate (*RC*) for A–10M, A–14M and A–NM transformation paths. The horizontal solid lines correspond to the lattice parameters of austenite. See Fig. 3 for the definitions of *γ*_2*M*_ and *a*_*C*_, *b*_*C*_, *c*_*C*_.

The evolution of the lattice parameters along the different transformation paths is summarized in Fig. 4. The monoclinic angle *γ*_2*M*_ (marked in Fig. 3) as a function of *RC* is shown in Fig. 4(a) and the lattice parameters *a*_*C*_, *b*_*C*_, *c*_*C*_ in cubic coordinates are shown in Fig. 4(b). For the A–NM path, *γ*_2*M*_ linearly decreases, which corresponds to a pure tetragonal shear of the cubic lattice resulting in an elongation of the lattice parameter *c*_*C*_ and a contraction of the lattice parameter *a*_*C*_. A similar almost linear decrease can be found for the A–14M transformation, but with a much smaller steepness due to the presence of oppositely oriented nanotwins in the structure. The opposite shears in the double and pentuple layers partially cancel each other.

The A–10M path shows a different behavior. The angle *γ*_2*M*_ remains nearly 90° for *RC* < 0.4, which corresponds to a tetragonal symmetry of lattice, i.e., the parameters *a*_*C*_ and *b*_*C*_ remain equal and slightly increase. This corresponds to a small orthorhombic deformation in the 2M coordinates. The lattice is mainly distorted by modulation (Fig. 3(b)), similar to that in 6M premartensite (cf. Fig. 1(b)). For *RC* > 0.4, the angle *γ*_2*M*_ starts decreasing and the parameters *a*_*C*_ and *b*_*C*_ start to differ, i.e., the structure becomes monoclinic. The *b*_*C*_ remains constant or slightly decreases, whereas *a*_*C*_ increases. In contrast, for the A–14M transformation, the parameters *a*_*C*_ and *b*_*C*_ differ immediately from the beginning of the transformation, when the *b*_*C*_ slightly decreases whereas the *a*_*C*_ rapidly increases. The *c*_*C*_ lattice parameter decreases for both these transformations along the whole path, and does not differ significantly from the *a*_*C*_ lattice parameter along the transformation path to the NM structure.

## Discussion

To interpret the thermodynamic aspects of the phase transformation, it is useful to consider the nanotwin-like character of the modulated martensites. From the point of view of nanotwinning, all modulated martensites can be built using the tetragonal building blocks or 2M cells, Fig. 1. To include the influence of nanotwin boundaries and their interaction, multiple layers must be considered as the basic building units. The total energies of the different martensites at 0 K are then the result of competition between the negative contribution of the boundary–boundary interaction across the double layer^13^ and the positive contribution of the single nanotwin boundary^28^. In other words, the double layers are energetically favorable compared to the triple or pentuple layers.

The energy relations between the martensitic structures can be qualitatively understood as follows: The 6O structure built solely from triple layers shows the highest energy^28^, because the presence of distant nanotwin boundaries increases its energy. The negative contribution of the nanotwin double layer explains the lowest energy of the 4O structure, because it is formed only by these basic units^13,28^. The NM structure does not spend energy on nanotwin boundaries but simultaneously has no energy gain from the negative contribution of the nanotwin boundary interaction across double layer. The energies of the 14M and 10M structures are very close to the energy of the NM structure (Fig. 2(a)). As the 10M (double and triple layers) can be viewed as a combination of the 4O (double layers) and the 6O (triple layers) structures, its energy lies between them. Similarly, the 14M is close to a combination of the 4O and the NM, thus its energy is slightly lower than that of the NM.

Although the described energy contributions of the nanotwin boundaries and their interactions explain the stability of modulated structures 10M and 14M, considering only thermodynamic aspects does not provide good agreement with experimental results. The purely thermodynamical approach predicts the 4O structure as the ground state of stoichiometric Ni_2_MnGa. The kinetics based on the calculated MEPs clearly show that in contrast to the A–4O path, there is no barrier on the A–10M path and the austenite spontaneously transforms to the 10M structure at 0 K. The A–6M path shows almost the same initial gradient in energy as the A–10M, which may explain the experimental appearance of premartensite at a non-zero temperature. The 6M–10M transformation, (Fig. 2(b)), is the most favorable transformation from premartensite. Thus in any case the 10M phase–and not the 4O phase with the lowest total energy–is the ultimate product of the transformations. This corresponds to the experimentally observed sequence A–6M–10M. To investigate if the 10M structure can further transform easily to the 4O structure, we also calculated the 10M–4O transformation path. We found a high energy barrier on the order of 2 meV/atom on this path, which explains why 10M is metastable relative to the lowest energy 4O phase.

The barrierless transformation to 10M can be understood using the geometry of crystal structures. The periodic distortion comprising alternating double and triple layers gradually increases with increasing RC. The appearance of triple layers at the beginning of the transformation (*RC* < 0.40) is related to the Fermi surface nesting and softening of the TA_2_ [ξξ0] phonon branch at *ξ* ≈ 0.33^6–9^. This soft mode is responsible for the initial shuffling of the (110) planes (see Fig. 3(b)), which is reflected in the structure as a small change in the lattice parameters and the corresponding tetragonal distortion (see Fig. 4(b)). A tetragonal distortion of austenite and shuffling of (110) planes can be seen also during the barrierless transformation to the cubic 6M structure comprising only triple layers. Both barrierless transformations involve triple layers, which indicate that they are an important part of the kinetics. Thus, the barrierless transformations are only possible with the occurrence of triple layers, in spite of the fact that double layers are preferred thermodynamically.

The increasing tetragonal distortion of the basic building blocks (referred to from now on as *tetragonalization*, see also Fig. 1) driven by the Jahn–Teller effect dominates the later part of the A–10M transformation (*RC* > 0.4, see Fig. 3(c) and (d)). Due to asymmetry originating from alternating double and triple layers, the tetragonalization results in monoclinic distortion of the structure (Fig. 4(a)). This is seen as a splitting of the *a*_*C*_ and *b*_*C*_ lattice parameters in Fig. 4(b).

The tetragonalization during the 6M–6O transformation does not result in monoclinic distortion, because there is a symmetrical arrangement of the triple layers. In the 6M–10M transformation, the double layers need to form within the initial triple layered structure. Interestingly, this complex rearrangement of layers involves a smaller energy barrier than the further progression to the 6O structure (Fig. 2(b)) by the tetragonalization of the building blocks.

To get an insight into the transformation process, we compare our calculation with experimental measurement of the elastic properties^29,30^. The stoichiometric Ni_2_MnGa shows the premartensitic transformation A → 6M, which is preceded by a gradual softening of the elastic moduli *C′* with decreasing temperature. The *C′* is linked with the TA_2_ [ξξ0] phonon branch and therefore the transformation will be realized by tetragonal lattice distortion. Indeed, as we showed above, the calculated A–6M path as well as the initial part of the A–10M path both involve a small tetragonal distortion of the austenitic lattice. A further decrease of temperature results in a gradual *C′* hardening of the 6M premartensite and a sharp *C*_44_ softening in the vicinity of the martensitic transformation. The *C*_44_ is linked with a monoclinic deformation, which dominates the 6M → 10M transformation, resulting in a monoclinic symmetry of the final 10M lattice. Again, this correlates well with the calculated monoclinic distortion dominating the later part of the A–10M (*RC* > 0.4) and 6M–10M transformation paths. On the other hand, the hypothetical calculated 6M–6O path is accompanied by further tetragonal deformation, which would require further softening of *C′*, contrary to experiment^30^. Thus, our geometrical interpretation of the most energetically favorable paths, A–10M and A–6M–10M, agrees with the experimentally observed development of the elastic constants near the martensitic transformation temperature. In short, at first the tetragonal distortion dominates (*C′* softening) followed by monoclinic distortion (*C*_44_ softening).

Using cubic coordinates the experimentally observed 10M martensite exhibits an almost tetragonal symmetry with much smaller monoclinic angle and smaller difference between the *a*_*C*_ and *b*_*C*_ lattice parameters than predicted by our calculations. We can identify two reasons for this discrepancy. At first, the *ab initio* calculations usually overestimate the Jahn–Teller effect and tetragonal ratio of the basic building blocks, which subsequently increases the monoclinicity of the 10M structure^21^. Secondly, the experimentally observed structure of 10M is not yet precisely determined despite intensive effort^31–35^. The determination of the structure is complicated by deep hierarchical twinning and other lattice imperfections^35^. For example, the 10M monoclinic structure can become apparently tetragonal by the {110} twinning^25,36^ and also the experimentally determined monoclinic angle can be smaller. To reveal the effect of such twining on the MEP, we also calculated the transformation between austenite and martensitic structure described by the ${(3\bar{2}2\bar{3})}_{1}$ stacking sequence, which represents the 10M martensite with the highest possible density of {110} twin boundaries. The MEP of this transformation does not show significant differences from the MEP in the A–10M transformation in Fig. 2(a). Thus, our calculation is not significantly affected by the presence of {110} twinning. The effect of other types of twining could be more significant and can modify existing barriers. This, however, does not change our finding that A–10M transformation will proceed spontaneously at 0 K.

Unlike the A–10M path, the A–14M transformation exhibits no tetragonal distortion, only monoclinic distortion, along the whole path (see Fig. 4(a)). This distortion arises again from the tetragonalization of the basic building blocks within the asymmetric geometry of the stacking sequence in the 14M structure. Since the structure now comprises pentuple layers instead of triple layers, the monoclinic distortion is larger than for the 10M. The *b*_*C*_ lattice parameter of the final 14M structure is smaller than the lattice constant of austenite *a*_*A*_, whereas *a*_*C*_ is significantly larger (see Fig. 4(b)). For a hypothetical path with tetragonal distortion in the initial phase of transformation, *b*_*C*_ would have to expand with the same steepness as *a*_*C*_. In the later phase, the fully relaxed double layer could be obtained only by a sharp contraction of *a*_*C*_ below the value corresponding to *a*_*A*_. The expansion of *a*_*C*_ followed by such a sharp contraction is apparently less energetically favorable than the direct monoclinic path, which exhibits only a tiny barrier in the beginning. To obtain a completely barrierless path involving pentuple layers, the softening of the TA_2_ branch should appear at ξ = 0.4, as was predicted for the alloy with an increased number of electrons^9^. In reality, an A → 14M transformation has been reported for an Ni-Mn-Ga alloy with an excess of Mn larger than 4 at. %^37^.

There is basically no geometrical reason why austenite could not transform directly to the 4O structure comprising only double layers, because *a*_*C*_ and *b*_*C*_ simultaneously increase along whole path with the same gradient (not shown) as in the beginning of the A–10M transformation. However, the absence of triple layers makes this transformation unfavorable, because it cannot energetically profit in the beginning of the transformation from the (101) plane shuffling corresponding to the TA_2_ branch soft mode and a barrier exists here. Similarly, there is a much larger barrier along the A–NM transformation, where there is no (101) plane shuffling at all. However, if the composition of the alloy is changed to be far from stoichiometry, this barrier vanishes due to the further softening of the TA_2_ branch at the Γ-point, resulting in a pure elastic instability^9,38^. Thus, the austenite can spontaneously transform to the NM martensite along a barrierless path for tetragonal distortion. Such barrierless paths were already shown for an alloy with excess Mn^39^ and for an alloy doped by Cu atoms in a Ga sublattice^40^. Such an A → NM transformation will be also preceded by a softening of the *C′* elastic moduli above the martensitic transformation temperature, although the transformation is driven solely by the Jahn–Teller effect and its mechanism completely differs from the mechanism of the A → 6M transformation. This behavior has recently been observed experimentally in thin films^41^ and polycrystalline bulk^42^.

To understand the martensitic transformations also at non-zero temperatures, the contributions of the magnetic excitations and phonon vibrations to the free energy have to be taken into account^14,43^. Although a full investigation of the free energies along the described paths is beyond the scope of this study, previous theoretical investigations have shown that both contributions stabilize the austenite with respect to martensite^16,44^. If we assume that the decreasing of the free energies due to these contributions is similar in all kinds of martensites and significantly smaller than in austenite, the barrier along each path will grow with increasing temperature. Because the A–10M transformation is barrierless at 0 K, at elevated temperature it is expected to exhibit also no barrier or the lowest barrier out of all the considered transformations. The recent theoretical prediction of the phase diagram shows that there is an interval of stability of 10M martensite below the martensitic transformation temperature *T*_*M*_^16^. However, that study does not include the 4O structure and 14M martensite had been predicted as the most stable at low temperature. The thermodynamic stability of 10M martensite near below *T*_*M*_^16^ and the stability of 6M premartensite near above *T*_*M*_^14^ also indicate that triple layers are thermodynamically preferred around *T*_*M*_, whereas double layers are preferred at low temperatures. Therefore, the stable 10M structure below T_M_ can be viewed as the result of the interplay between triple layers and double layers.

## Conclusions

Our *ab initio* investigation of martensitic transformations shows that beside the thermodynamic aspects, the kinetic aspects represented by energy barriers along the transformation paths also have to be taken into account for a full understanding of the modulated martensitic structures in Ni_2_MnGa compound. The modulated structures are thermodynamically stabilized by the nanotwin boundary–boundary interaction across the double layers, which results in the lowest total energy of the 4O structure. On the other hand, the transformation paths from austenite to the 4O, 14M or NM martensites exhibit energy barriers, whereas there is no barrier along the transformation paths to 10M martensite and 6M premartensite. The smallest barrier can be found for subsequent 6M–10M transformation compared to the transformations from 6M to other phases and thus the transformation to 10M is always preferred. Initially, this transformation is driven by a softening of the TA_2_ [ξξ0] phonon branch at *ξ* ≈ 0.33, corresponding to the shift of the (110) planes in forming triple layers. In the later stage, the transformation evolves by tetragonalization of the basic building blocks due to the Jahn–Teller effect and the formation of oppositely oriented double layers beside the triple layers to decrease the total energy of the structure. Therefore, the final 10M structure is determined not by competition but by the subsequent complementary action of phonon softening and the Jahn–Teller effect. The calculated path of the transformation agrees well with the experimentally observed evolution of the elastic constants and the final observed 10M phase.

## Methods

All computations were performed by applying the plane-wave based spin-polarized DFT method with the Vienna Ab Initio Simulation Package^45,46^. The electron ion interaction was described with the projector augmented wave method^47,48^. The electron exchange and correlation energy were treated within the generalized gradient approximation in the Perdew–Burke–Ernzerhof formalism^49^. The cut-off energy of 600 eV and Methfessel–Paxton electron smearing method^50^ with *σ* = 0.02 eV were used. The Brillouin zone (BZ) was sampled using a Γ-point centered mesh with the smallest allowed spacing between *k*-points equal to 0.1 Å^−1^. The optimization of the geometry was performed when the convergence criterion on the forces became smaller than 1 meV ⋅ Å^−1^ and the energy difference was smaller than 10^−6^ eV. The effect of spin-orbit coupling was not included for their small contribution on the calculated energies. In our approach, while searching for the minimum energy path between the austenite and martensites, the path was linearly interpolated by ten images and then each image relaxed by the G-SSNEB procedure^17^ with respect to the pathway described by reaction coordinate (*RC*). The reaction coordinate, which effectively represents the complex change in 3*N*-dimensional coordinate space including all atomic and lattice degrees of freedom, is defined as an accumulated distance from the initial state to the *i*-th image, normalized by the sum of distances between all images. The distances between images are calculated according to regular G-SSNEB scheme^17^. Thus, the starting point of transformation in austenite corresponds to *RC* = 0 and the final point in fully transformed martensite corresponds to *RC* = 1. The snapshots of structure along the A–10M transformation path shown in Fig. 3 were obtained using the Atomic Simulation Environment^51^.
